# Revisiting Colon Cancer Progression: A Containment-Based Conceptual Framework

**DOI:** 10.3390/life16040679

**Published:** 2026-04-16

**Authors:** Roxana Loriana Negrut, Adrian Cote, Adrian Marius Maghiar

**Affiliations:** Department of Surgical Disciplines, Faculty of Medicine and Pharmacy, University of Oradea, 410073 Oradea, Romania; amaghiar@uoradea.ro

**Keywords:** colon cancer, tumor progression, anatomical containment

## Abstract

Patterns of colon cancer recurrence demonstrate a high degree of anatomical reproducibility, consistently aligning with mesofascial planes and compartmentalized vascular and lymphatic territories, as evidenced by pathological, surgical and imaging studies. These frameworks describe recognized routes of spread but do not provide an integrated anatomical explanation for understanding why tumor progression often aligns with mesofascial planes, embryological boundaries and cavity-specific niches, nor for why preservation of structural integrity during surgery is associated with improved oncological outcomes. This work proposes a spatial containment model of colon cancer progression, in which tumor dissemination reflects sequential breaches of anatomically defined barrier systems. The Colon Cancer Containment System is proposed as a three-tier framework in which tumor progression reflects sequential breaches of containment at the tissue (microcontainment), mesenteric (mesocontainment) and peritoneal or systemic (macrocontainment) levels. At each stage, anatomical structures function as barrier systems that constrain tumor spread and shape directionality of progression. Disruption of these barriers, whether tumor-driven or iatrogenic, is associated with relatively consistent patterns of local, regional, and distant recurrence. Within this approach, established prognostic features such as tumor–node–metastasis (TNM) stage, extramural vascular invasion, perineural invasion and margin status may also be interpreted as markers of containment integrity, in addition to their established roles as indicators of tumor aggressiveness. Surgical plane preservation is reframed as a biologically meaningful act of containment maintenance. By organizing validated observations within an anatomically patterned architecture, the containment framework provides a coherent model for interpreting reproducible recurrence patterns and clarifies the biological significance of surgical integrity. This perspective complements existing oncological paradigms, supports anatomically informed risk stratification and generates testable hypotheses for future clinical and translational research.

## 1. Introduction

Current models of colon cancer dissemination describe lymphatic, vascular and peritoneal spread as distinct routes of progression, which are typically addressed as parallel processes rather than as components of an integrated anatomical system [1,2,3]. While these pathways are individually well characterized, they fail to explain a fundamental and consistently observed clinical reality: recurrence patterns are not random. Local, regional and distant failures repeatedly align with mesofascial planes, embryological boundaries and cavity-specific niches [4,5,6]. Within this framework, containment failure refers to loss of anatomical barrier integrity, whereas recurrence represents its clinical manifestation. The discrepancy between theoretical dissemination routes and real-world recurrence maps has been recognized for decades, yet has remained insufficiently addressed. This article does not propose anatomical containment as an alternative to tumor biology, but as a complementary spatial framework through which biological aggressiveness becomes clinically manifest.

This gap reflects an anatomically incomplete understanding of tumor progression. Existing models describe how tumor cells may disseminate but do not account for why progression follows reproducible spatial patterns or why disruption of specific anatomical structures exerts a measurable influence on oncological outcomes [7,8]. Consequently, recurrence has often been interpreted as a probabilistic event driven predominantly by tumor biology, with anatomical structure largely considered a contextual background rather than an active determinant of progression.

Advances in mesenteric anatomy and plane-based surgical techniques have challenged this view [4,9,10]. Accumulating evidence indicates that preservation of structural integrity during resection is associated with improved oncological outcomes [5,11]. This observation carries a direct implication: anatomical containment is not a technical artifact but a biological reality. If maintenance of mesenteric and fascial integrity alters prognosis, tumor progression must be constrained by structural barriers whose breach modifies disease behavior.

Fragmented anatomical reasoning has, in turn, contributed to fragmented oncological interpretation. Lymphatic spread, vascular invasion and peritoneal dissemination have largely been considered independent mechanisms, obscuring the structural logic that governs their interaction. In the absence of a unifying anatomical framework, validated pathological and surgical observations remain conceptually isolated and clinically under-integrated.

This article introduces the Colon Cancer Containment System, a three-tier conceptual framework in which tumor progression reflects sequential breaches of anatomical containment at the tissue (microcontainment), mesenteric (mesocontainment) and peritoneal and systemic (macrocontainment) levels. Rather than extending existing dissemination models, the approach reorganizes established evidence within an anatomically grounded architecture, reconciling recurrence patterns, pathological features and surgical outcomes.

While the proposed containment framework shares conceptual overlap with established surgical paradigms such as complete mesocolic excision and compartment-based surgery, its objective and scope are different. Existing approaches primarily describe surgical strategy and anatomical planes to optimize oncological resection, emphasizing preservation of mesenteric integrity and en bloc removal of the tumor-bearing compartments.

In contrast, the Colon Cancer Containment System is an explanatory conceptual model that integrates anatomical, pathological and clinical observations across all stages of disease progression. It conceptualizes tumor progression as a hierarchical process of containment breach, linking early tissue-level invasion, mesenteric dissemination, and peritoneal or systemic spread within a unified spatial model. This distinction allows the model to extend beyond operative anatomy, providing a conceptual basis for interpreting recurrence patterns, prognostic markers, and dissemination pathways as interconnected manifestations of structured containment failure rather than as independent or parallel processes.

By reframing validated observations through a containment-based lens, this article proposes a unified structure that may help explain reproducible spatial patterns of colon cancer progression and how loss of anatomical integrity is associated with alterations in disease trajectory. Interpretations inconsistent with these anatomical constraints are reconsidered within a structurally grounded framework.

The novelty of this model lies not in the identification of new dissemination routes, but in the reorganization of established anatomical, pathological and surgical observations into a hierarchical containment architecture that explains their spatial coherence.

## 2. Materials and Methods

This work presents a hypothesis-generating conceptual framework based on the integration of established anatomical, embryological, pathological and surgical evidence relevant to colon cancer progression. Representative literature was used to support the proposed model and to illustrate reproducible patterns of tumor spread and recurrence. Relevant publications were identified primarily through Web of Science and PubMed, with emphasis on studies addressing colon cancer anatomy, mesenteric and mesofascial organization, dissemination pathways and recurrence patterns, surgical plane integrity, peritoneal metastasis and tumor microenvironmental interactions. The articles reviewed included both foundational and contemporary studies, with emphasis on work published over the past two decades, while also incorporating earlier contributions in surgical and oncological theory where relevant.

Literature selection was purposive and guided by relevance to spatial aspects of tumor progression and containment, prioritizing studies that provide anatomical, pathological, surgical or translational insights into mechanisms of local invasion, regional spread, and distant dissemination.

The figure was developed using Python (Anaconda Navigator 2.7.0, JupyterLab, Austin, TX, USA).

The aim of this conceptual synthesis was to integrate conceptually informative evidence into a coherent hierarchical containment model of colon cancer progression.

## 3. Conceptual Framework

### 3.1. Definitions of Containment Compartments

Within the Colon Cancer Containment System, tumor progression is organized across three anatomically defined containment compartments. These compartments are distinguished by scale, structural composition and biological function, and are defined below.

**Microcontainment** refers to tissue-level anatomical constraints operating within and immediately adjacent to the primary tumor compartment. It encompasses stromal architecture, extracellular matrix organization, mechanical anisotropy and the spatial distribution of vascular and neural structures. Microcontainment governs early tumor extension and determines the directionality of initial invasive spread through structurally permissive interfaces.

**Mesocontainment** denotes the intermediate containment level constituted by the mesentery as an organized barrier system. It includes mesenteric fascial envelopes, mesofascial planes, embryological fusion boundaries and compartmentalized vascular and lymphatic territories. Mesocontainment constrains regional tumor progression and channels dissemination along anatomically predetermined pathways until its integrity is breached.

**Macrocontainment** describes containment operating at the level of the peritoneal cavity and systemic circulation. It encompasses peritoneal surface integrity, cavity-level organization, fluid dynamics and physiological conditions influencing tumor cell implantation and survival. Failure of macrocontainment permits cavity-wide and systemic exposure, shaping the distribution of peritoneal and distant disease.

In contrast to micro- and mesocontainment, which correspond to more clearly defined anatomical structures, macrocontainment represents a broader and less discretely bounded systemic domain, in which dissemination is influenced not only by anatomical exposure but also by tumor-host interactions, immune dynamics and other systemic factors. As such, macrocontainment should be interpreted as an integrative systemic domain rather than a compartment.

These compartments are not independent mechanisms of spread but sequentially related containment layers. Breach at one level alters exposure and vulnerability at the next, contributing to anatomically reproducible patterns of progression and recurrence.

For clarity, the hierarchical organization of the Containment System and potential application of the model, representative clinical, pathological and anatomical markers corresponding to each containment level are summarized in Table 1. These examples are intended to illustrate how the concept of containment breach may be operationalized in clinical and research contexts, rather than to define strict criteria.

**Figure 1:**
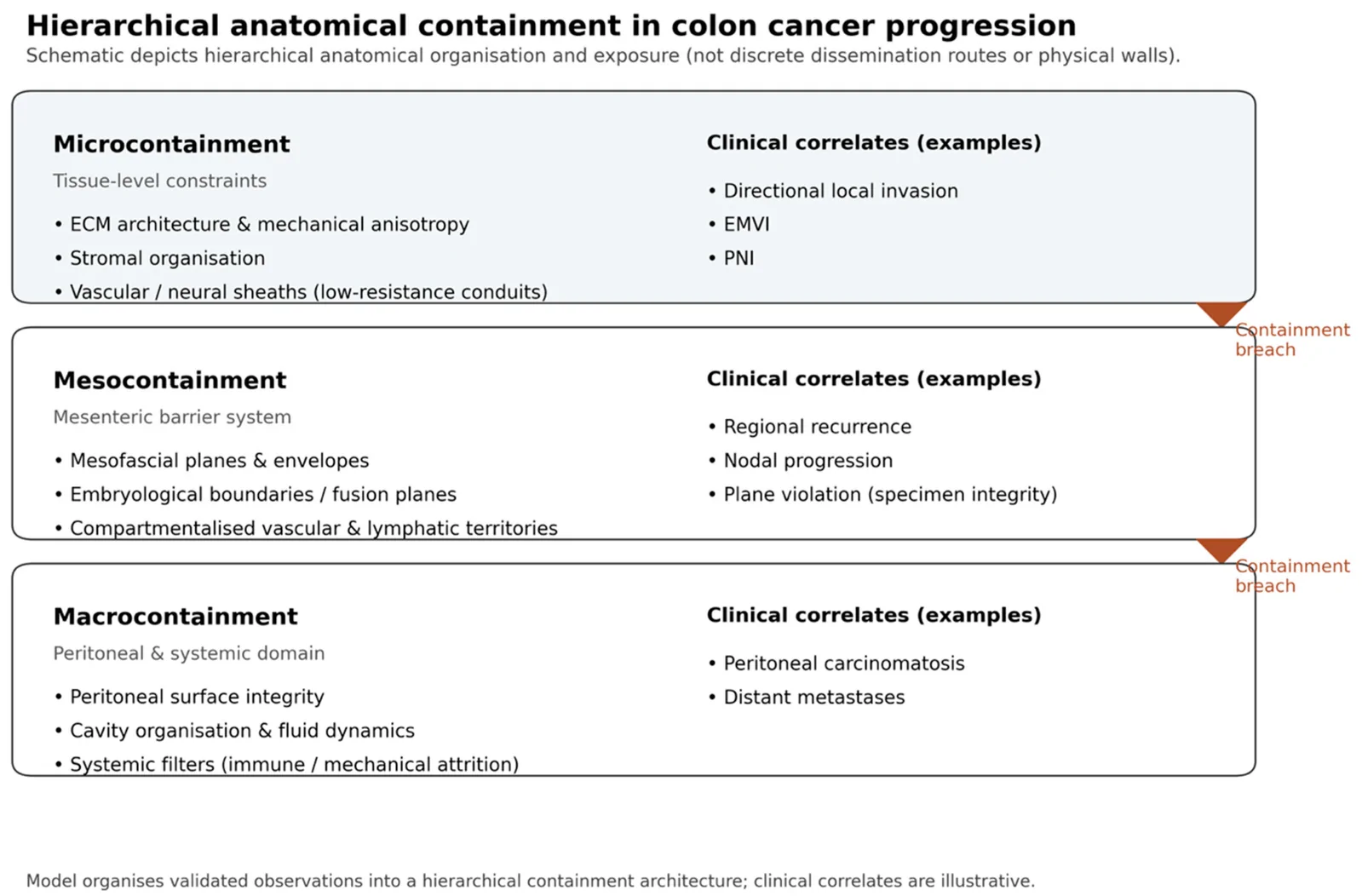
Schematic representation of the Colon Cancer Containment System. The figure illustrates the three-tier structure of the anatomical containment that governs colon cancer progression. Microcontainment represents tissue-level constraints, including stromal architecture, extracellular matrix (ECM) organization and vascular and neural interfaces that influence early tumor invasion. The intermediate layer, mesocontainment, corresponds to the mesentery, including mesofascial planes and the compartmentalized vascular and lymphatic territories that constrain regional spread. Macrocontainment represents the level of peritoneal cavity and systemic circulation, which includes peritoneal integrity, fluid dynamics and systemic constraints that influence peritoneal and distant metastasis. Abbreviations: ECM, extracellular matrix; EMVI, extramural vascular invasion; PNI, perineural invasion.

To aid conceptual orientation, the hierarchical containment framework is illustrated in Figure 1.

### 3.2. Microcontainment: Tissue-Level Constraints and Early Breaches

Microcontainment represents the first anatomical level at which colon cancer progression is constrained by local tissue architecture. At this scale, tumor expansion is shaped by the organization of the extracellular matrix, stromal cellular composition, mechanical anisotropy and the spatial distribution of vascular and neural structures. Together, these elements define zones of resistance and vulnerability that influence the direction and mode of early tumor spread.

Microcontainment is conceptualized as an anatomically grounded but biologically modulated domain, in which structural barriers such as extracellular matrix organization and stromal architecture interact with tumor cell-intrinsic and microenvironmental factors that regulate invasion and local progression.

The colonic wall and adjacent stromal compartment are mechanically heterogeneous rather than uniform. Collagen fiber orientation, fibroblastic alignment and regional differences in tissue stiffness create anisotropic environments that favor directional invasion along paths of least resistance [12,13,14]. Tumor cells therefore tend to extend along pre-existing structural interfaces rather than infiltrating tissue uniformly [12,15]. As a result, early progression follows structured microanatomical trajectories that precede overt regional or systemic dissemination.

Within this structural context, extramural vascular invasion and perineural invasion can be interpreted as specific microcontainment breaches rather than as nonspecific indicators of aggressive tumor biology. Vascular and neural sheaths act as longitudinal conduits traversing tissue compartments and provide low-resistance pathways once surrounding containment is compromised [12,16,17,18]. The consistent association of these features with adverse outcomes reflects their role in enabling structured escape from the primary tumor compartment [5,18].

At the microcontainment level, disruption of local barriers reflects not only structural vulnerability but also the influence of intracellular signaling pathways that regulate tumor cell proliferation, survival and invasion [19].

Local inflammatory states and tumor–stroma interactions further modulate microcontainment integrity. Inflammatory microzones influence matrix remodeling, vascular permeability and immune surveillance, thereby increasing susceptibility at specific tissue interfaces [16,20,21,22]. Microbial and immune gradients within the tumor microenvironment may accentuate local vulnerability, acting as modifiers of containment strength rather than independent determinants of invasion direction [22].

Importantly, failure of microcontainment does not imply immediate systemic exposure. Early breaches typically remain spatially constrained at the tissue and mesenteric interface, directing tumor cells toward anatomically connected compartments rather than enabling diffuse dissemination. This may help explain why features such as extramural vascular invasion and perineural invasion are strong predictors of recurrence while still producing patterned, rather than random, progression [5,18].

By reframing early invasive events as containment breaches occurring at structurally permissive interfaces, microcontainment provides a spatially grounded link between histopathological features and reproducible patterns of progression. This perspective shifts interpretation of early invasion from a purely biological descriptor to an anatomically contextualized event with downstream implications for regional and cavity-level disease evolution.

### 3.3. Mesocontainment: The Mesentery as an Organized Barrier System, Not Surgical Packaging

Within the containment structure, the mesentery is understood not as a passive carrier of vessels and lymphatics but as an organized anatomical barrier system that governs regional tumor progression. Its structure reflects embryological development and is defined by layered fascial envelopes, mesofascial crescents and embryological fusion planes that persist into adult anatomy. Together, these elements establish structured pathways along which tumor spread is channeled once mesocontainment integrity is breached [4,9,10].

The mesentery therefore does not represent a neutral background for disease extension. Its compartmentalized organization constrains tumor progression and imposes directionality on regional dissemination. Breach of specific mesofascial layers exposes predefined routes rather than permitting indiscriminate spread, accounting for the reproducibility of regional recurrence patterns across patients and surgical approaches [4,11,23].

Within this system, vascular territories and lymphatic basins function as integrated anatomical compartments rather than independent conduits. Access to these territories typically occurs in an ordered sequence following loss of mesenteric containment integrity [1,4]. From this perspective, lymphatic spread is reframed not as a parallel mechanism of dissemination but as a consequence of anatomically governed barrier failure.

The interface between the mesentery and the peritoneum represents a critical transition zone within the containment hierarchy. At this junction, fascial continuity is attenuated and containment strength diminishes, creating a consistent point of relative vulnerability. Breach at this interface facilitates exposure to the peritoneal cavity and marks the transition from regionally constrained progression to macrocontainment failure, with implications for peritoneal and systemic dissemination [4,6,24,25].

Importantly, many recurrence clusters previously described as “unexpected” may be reinterpreted within an anatomical context once specific mesocontainment layers are violated [4,23]. Rather than reflecting stochastic tumor behavior, these patterns arise from the structured organization of the mesentery and the sequential exposure of downstream compartments. Recognizing the mesentery as an organized barrier system therefore provides a coherent explanation for interpreting regional progression and reinforces its role as a central determinant of oncological behavior rather than a surgical afterthought.

### 3.4. Macrocontainment: Peritoneal and Systemic Exposure Appears to Follow Structured Constraints

Failure of macrocontainment marks the transition from anatomically constrained disease to cavity-level and systemic exposure. Within the containment framework, this transition can be understood as being influenced by structural and biological constraints that show reproducible patterns.

In colon cancer, macrocontainment failure most commonly manifests initially at the peritoneal level, with systemic dissemination frequently occurring downstream or in parallel, depending on tumor location, vascular access and host factors.

Peritoneal surfaces exhibit differential susceptibility to tumor implantation based on mesothelial integrity, microvilli orientation and local immune tone [6,26,27,28]. These surface-specific characteristics influence tumor cell adhesion, survival and proliferation, creating spatially patterned vulnerability across the peritoneal cavity. Consequently, peritoneal involvement follows consistent anatomical distributions rather than diffuse or random spread.

Intra-abdominal fluid dynamics further shape macrocontainment failure. Gravity-dependent fluid movement, diaphragmatic motion and compartmentalized peritoneal fluid circulation generate hydrodynamic settlement zones within the peritoneal cavity where liberated tumor cells preferentially accumulate [29,30]. These stagnation points act as ecological niches that facilitate implantation, accounting for the recurrent localization of peritoneal metastases at specific cavity sites [6,31,32].

Postoperative physiological changes may transiently amplify vulnerability at the macrocontainment level. Surgical stress induces short-term immune modulation, metabolic shifts and inflammatory responses that can reduce resistance to tumor cell implantation. The early postoperative period therefore represents a biologically unstable environment in which previously contained tumor cells may exploit newly permissive peritoneal and systemic conditions during this window [33,34].

Importantly, macroscopic patterns of peritoneal dissemination mirror cavity organization rather than reflecting tumor aggressiveness in isolation [6,29]. Within the containment approach, extensive peritoneal disease is interpreted as the combined consequence of upstream containment breaches and cavity-level permissiveness, rather than as an inherent property of tumor biology.

At the macrocontainment level, anatomical exposure represents only one component of dissemination, as successful metastatic colonization also depends on tumor-host interactions, including immune surveillance, immune evasion and systemic microenvironmental compatibility [35].

Systemic dissemination represents a distinct domain of macrocontainment failure rather than a stochastic extension of tumor aggressiveness. Entry into the systemic circulation does not confer automatic metastatic success. Circulating tumor cells are subject to substantial attrition through mechanical stress, immune surveillance and failure to colonize permissive secondary niches. Organ-specific vascular architecture, immune microenvironments and stromal compatibility impose additional constraints that shape the distribution of distant metastases [3,7]. Within the containment framework, hematogenous spread is therefore understood as a structured process governed by sequential constraints, in which loss of mesenteric and cavity-level containment permits exposure to systemic filters rather than unrestricted dissemination.

By framing peritoneal and systemic dissemination as structured outcomes of macrocontainment failure, this model integrates anatomical organization with physiological context. It provides a coherent explanation for why advanced disease follows recognizable spatial patterns and reinforces the concept that late-stage progression remains governed by structural constraints rather than chance.

The containment levels described in this framework are not intended to represent mutually exclusive or discretely classifiable categories. They reflect overlapping and interacting domains of disease progression, which may coexist within the same patient. As such, the model is not designed as a staging system, but as an interpretative structure through which diverse clinical and pathological findings may be integrated across spatial scales.

### 3.5. Containment Failure Patterns: Mapping Routes of Progression

Patterns of colon cancer recurrence demonstrate a high degree of anatomical fidelity that is not fully explained by stage-based or probabilistic models alone [5,6,29]. Local, regional and distant failures consistently map onto distinct containment layers, reflecting the level and location of preceding barrier breach. This observation challenges the assumption that recurrence is primarily a random event driven by tumor biology in isolation.

Local recurrence may reflect failure at the level of microcontainment. Tissue-level barrier compromise occurs at anatomically permissive interfaces and may influence the direction of residual tumor growth along pre-existing structural planes rather than producing diffuse regrowth. As a result, early recurrence follows reproducible local trajectories rather than entirely random spatial distribution [5,11,24].

Regional recurrence aligns closely with loss of mesocontainment integrity. Violation of mesocolic planes, disruption of fascial envelopes and breach of embryological boundaries permit access to regional vascular and lymphatic compartments [4,11,24]. These events are often categorized as “technical failures” in postoperative assessment; however, within the containment framework they may be interpreted as anatomically localized breaches that contribute to structured patterns of disease spread. In this context, intraoperative factors such as traction-related tears, thermal microperforations and improper mobilization may compromise mesenteric containment in a potentially biologically meaningful manner despite limited macroscopic visibility [36,37]. The extent to which such micro-injuries influence oncological outcomes remains uncertain and should be considered hypothesis-generating, requiring validation in prospective studies.

Distant and peritoneal recurrence patterns further illustrate the role of anatomical constraint. Peritoneal carcinomatosis does not distribute uniformly throughout the abdominal cavity but preferentially involves sites corresponding to hydrodynamic stagnation zones and transition-point vulnerability. These recurrence clusters reflect cavity-level organization and fluid dynamics rather than random implantation, supporting macrocontainment failure as a structured process [6,29,32].

By integrating these observations, the containment framework offers a complementary perspective to conventional staging systems. Tumor–node–metastasis classification quantifies disease extent but does not capture the spatial logic governing progression and failure. A containment-based perspective may help interpret why recurrences arise in specific anatomical locations, linking anatomically defined breach points to downstream disease patterns that statistical descriptors alone cannot resolve.

### 3.6. Surgical Integrity as Containment Preservation

Within a containment-based model, surgical integrity is defined by preservation of anatomical barriers rather than by the pursuit of visually pristine dissection planes. Mesocolic completeness is not a stylistic or aesthetic preference but represents the operational expression of maintaining mesocontainment integrity [4,5]. When embryological planes and fascial envelopes are respected, regional compartments remain intact, limiting downstream exposure and constraining tumor progression.

Loss of containment may occur before the specimen leaves the abdominal cavity. Excessive traction, thermal injury and inappropriate mobilization techniques can degrade mesenteric and fascial integrity at a microstructural level, creating biologically meaningful breaches that are not immediately apparent [5,24,36]. Within the containment framework, such events are interpreted as procedural contributors to barrier failure rather than minor technical imperfections.

Specimen handling constitutes a continuation of the containment assessment chain beyond in vivo resection. Disruption of mesenteric planes, fragmentation of fascial envelopes or loss of structural orientation during ex vivo handling may compromise containment that appeared preserved intraoperatively. Ex vivo handling does not contribute to tumor dissemination but may alter the apparent integrity of mesenteric planes and fascial envelopes during pathological assessment, thereby influencing interpretation of the degree of containment preservation achieved in vivo.

Importantly, plane-based surgery is reframed not as a marker of increased radicality but as an expression of restraint. By preserving natural anatomical boundaries and avoiding forced progression across anatomical compartments, surgical technique functions to maintain containment rather than to extend resection indiscriminately. This distinction clarifies why meticulous plane preservation is associated with improved oncological outcomes independent of resection extent [5,11].

By redefining surgical quality as containment preservation, the framework integrates operative conduct into the broader biology of colon cancer progression. Surgical integrity thus emerges as a modifiable determinant of anatomical stability, with implications not only for immediate resection success but also for the trajectory of subsequent disease behavior.

## 4. Discussion

The proposed framework is not intended to replace established explanations of tumor progression, including vascular and lymphatic dissemination pathways, organ tropism, immune modulation, or treatment-related effects. Rather, it provides a complementary spatial perspective through which these mechanisms may be interpreted within a structured anatomical context. In this sense, containment failure may be viewed as one of several interacting determinants of disease progression.

A key contribution of the proposed structure is its ability to integrate diverse clinical and pathological observations across multiple spatial levels of disease progression. The containment model provides a structured perspective, allowing features such as invasion patterns, mesenteric plane integrity and metastatic dissemination to be interpreted as interconnected manifestations of containment integrity or failure. In this way, the model does not replace existing explanations but offers an integrative framework through which their relationships may be more coherently understood. Rather than providing direct predictive capability, the model generates testable hypotheses regarding how multi-level containment disruption may relate to recurrence patterns and disease progression, thereby offering basis for future investigation.

### 4.1. Positioning the Colon Cancer Containment System Within Existing Frameworks

The proposed containment structure builds upon and integrates established surgical and anatomical paradigms, including complete mesocolic excision (CME) and surgical approaches based on embryological plans and compartmental anatomy, all of which emphasize the importance of anatomical planes and mesenteric interfaces in oncological outcomes. These approaches primarily define operative strategies and focus on the preservation of mesenteric integrity during resection. In contrast, the Colon Cancer Containment System extends this perspective by providing a broader explanatory framework that includes multiple levels of disease progression, from tissue-level invasion to systemic dissemination. This distinction allows the framework to provide a structured interpretation of recurrence patterns and dissemination pathways beyond the scope of existing surgical paradigms.

### 4.2. Clinical and Translational Implications: A Model That Reorganizes Oncologic Thinking

Interpreting colon cancer progression through a containment-based model has implications that extend beyond surgical technique, influencing oncological interpretation, risk stratification and translational strategy. By organizing disease behavior around the integrity of anatomical barriers, the framework provides a unifying logic that contextualizes established prognostic parameters within a coherent spatial hierarchy.

While the containment framework emphasizes anatomical structure as a determinant of spatial progression, tumor biology remains a critical modulator of how and when containment barriers are breached. Molecular and genetic heterogeneity, including variations in microsatellite instability, KRAS and BRAF mutations, and other regulatory pathways, may influence tumor invasiveness, interaction with the extracellular matrix, and the capacity to extend to vascular, neural and peritoneal interfaces [7,16,22]. Within this context, biological factors can be understood as modifying the integrity of the containment layers rather than replacing the structural constraints that shape disease distribution. The interplay between molecular drivers and anatomical organization may determine both the timing and extent of containment failure, suggesting that spatial and biological models of progression are complementary rather than mutually exclusive [7].

Genomic and molecular drivers may contribute to the ability of tumors to overcome biological constraints, while anatomical containment provides a complementary framework for understanding where and how this escape becomes spatially and clinically manifest. Emerging evidence on higher-order genomic regulation further supports this integrated view, emphasizing the role of complex regulatory architectures in shaping tumor progression [38]. These processes are also influenced by mutation-driven carcinogenesis and tumor suppressor instability, which contribute to tumor heterogeneity and interact with spatial constraints to shape disease behavior [39].

Tumor progression and recurrence patterns are influenced by multiple mechanisms, including vascular anatomy, lymphatic drainage pathways, clonal selection, organ tropism, immune modulation, treatment-related effects, and detection bias. The proposed containment framework is not intended to replace these explanations, but rather to provide a complementary spatial perspective through which they may be integrated. Within this context, the containment model does not redefine these processes but organizes them according to their relationship with anatomical barriers and compartmental integrity. This way, it offers a structured way to interpret how diverse biological and clinical factors may converge to produce reproducible spatial patterns of disease progression.

Within this model, conventional markers such as tumor–node–metastasis stage, extramural vascular invasion, perineural invasion and margin status are interpreted as indicators of containment integrity rather than as isolated expressions of tumor aggressiveness [18,40,41,42]. These parameters reflect the depth and level of anatomical barrier breach, clarifying their prognostic significance when situated within the sequential failure of micro-, meso- and macrocontainment.

A containment-based perspective also supports a more anatomically informed approach to recurrence risk stratification. Rather than viewing recurrence as a probabilistic outcome derived predominantly from tumor biology, the framework emphasizes the spatial logic of progression. When recurrence risk is assessed in relation to the level and location of containment failure, patterns of local, regional and distant relapse become more readily interpretable.

The model further implies that current diagnostic, operative and educational tools require conceptual alignment with containment principles. Existing imaging modalities, intraoperative mapping techniques and digital training platforms are optimized for tumor localization and resection planning but remain insufficiently sensitive to containment integrity. Development of containment-aware technologies capable of assessing fascial continuity, mesenteric plane preservation and transition-zone vulnerability represent a key translational opportunity.

The relationship between surgical plane integrity and oncological outcome is likely multifactorial and should be interpreted with caution. Preservation of mesenteric planes may reflect several overlapping phenomena: first, it may act as an indirect indicator of the underlying extent of disease and tumor aggressiveness; second, it may indicate surgical precision and overall perioperative quality; and third, it may contribute directly to containment preservation by limiting disruption of anatomical barriers. Within the present framework, these possibilities are not mutually exclusive. Surgical integrity is interpreted as one of several factors that may influence containment at the mesenteric level, interacting with tumor biology and host-related variables. The relative contribution of each mechanism remains uncertain and requires further investigation in appropriately designed studies.

From a research perspective, the containment structure defines a structured translational roadmap. This includes anatomical validation of containment layers across tumor locations, ecological characterization of permissive niches at containment interfaces and prospective mapping of failure points in relation to surgical and pathological variables. Such efforts would enable systematic testing of the framework’s assumptions and facilitate integration with molecular, immunological and microbiome-based insights.

By providing a shared anatomical language across surgery, pathology, radiology and oncology, the containment approach supports more coherent interpretation of disease behavior. This reorganization around structure rather than abstraction establishes a foundation for anatomically informed innovation in both clinical practice and translational research.

### 4.3. Limitations

This study presents a conceptual framework and does not include validation using prospective clinical datasets. The proposed model is hypothesis-generating and requires further evaluation in studies designed to assess its reproducibility, clinical applicability, and relationship to established prognostic factors.

Several limitations of the proposed containment framework should be acknowledged. First, the proposed model is conceptual and hypothesis-generating and it has not been validated using prospective clinical datasets. Second, the model simplifies complex biological and clinical processes into a structured representation, which may not fully capture the variability and heterogeneity observed in individual patients. Third, the boundaries between the containment levels are not strictly defined and may overlap in practice, reflecting the continuous dynamic nature of tumor progression. The framework does not aim to provide predictive capability or direct clinical decision-making criteria, and its applicability requires further validation in future studies designed to assess its reproducibility and clinical relevance.

Tumor heterogeneity influences the relative contribution of micro-, meso- and macrocontainment across patients and tumor subtypes. This variability may modulate the sequence and prominence of containment failures and highlights the need for cautious interpretation of the model at the individual level.

Surgical technique remains inherently variable and incompletely quantifiable. Differences in traction, dissection strategy, thermal exposure and mesenteric handling may obscure distinctions between intrinsic tumor-driven containment breaches and iatrogenic disruption. As a result, causal attribution between specific containment failures and oncological outcomes remains challenging, particularly in retrospective analyses.

Current imaging and pathological assessment lack sufficient spatial resolution to identify microcontainment failure in real time. Fascial continuity, microstructural disruption and early barrier compromise are not routinely visualized or reported in a standardized manner, limiting prospective stratification based on containment status.

At the level of macrocontainment, peritoneal fluid dynamics and postoperative systemic changes remain incompletely characterized. Although experimental and clinical observations suggest that cavity-level biology and early postoperative conditions influence tumor implantation, these processes are not yet fully modeled or integrated into predictive frameworks.

Finally, prospective validation is required across tumor locations, surgical approaches and institutional settings. The containment approach is intended as a hypothesis-generating construct that organizes existing observations and guides future study design, rather than as a definitive predictive tool.

## 5. Conclusions

The Colon Cancer Containment System reframes tumor progression as a hierarchical sequence of anatomically constrained barrier failures rather than as a collection of independent dissemination routes. By integrating tissue-level architecture, mesenteric organization and cavity-level biology into a unified framework, the model provides a coherent framework explanation for interpreting the reproducible patterns of recurrence observed in clinical practice.

This containment-based perspective does not replace existing molecular or staging paradigms but complements them by embedding biological behavior within a spatial and structural context. It clarifies the significance of established pathological features, reconciles surgical plane integrity with oncological outcomes and highlights anatomy as an active determinant of disease evolution rather than a passive background.

By positioning surgical intervention as a modulator of containment rather than solely a means of tumor removal, the framework offers a biologically grounded rationale for the impact of operative quality on recurrence. At the same time, it generates testable hypotheses that may inform future imaging strategies, pathological reporting and translational research aimed at linking structure, biology and outcome.

Cancer progression reflects the interaction of structured and stochastic processes rather than a purely deterministic or purely random phenomenon. While anatomical organization may impose spatial constraints that influence pathways of dissemination, tumor behavior is also shaped by stochastic elements, including clonal evolution, microenvironmental variability and treatment-related effects. Within this context, the proposed containment model is not intended to reduce tumor progression to a strictly structured process, but to highlight how anatomical constraints may interact with biological variability to produce patterns that are neither entirely random nor fully predictable. This perspective emphasizes complementarity rather than opposition between structural and stochastic determinants of disease progression.

## Figures and Tables

**Table 1:** Colon Cancer Containment System: hierarchical anatomical compartments and patterns of failure.

Containment Level	Anatomical Scale	Principal Structural Barriers	Containment Failure	Clinical Manifestations
Microcontainment	Tissue and peritumoral stroma	Extracellular matrix architecture Stromal organization Vascular and neural sheaths	Local barrier at structurally permissive interfaces	Directional local invasion Extramural vascular invasion (EMVI) Perineural invasion (PNI); Depth of invasion
Mesocontainment	Mesentery and regional compartments	Mesenteric fascial envelopes Mesofascial planes Embryological fusion boundaries Vascular and lymphatic territories	Breach in mesenteric planes and compartmental integrity	Regional and loco-regional recurrence, mesocolic plane integrity; specimen quality grading; circumferential resection margin (CRM)
Macrocontainment	Peritoneal cavity and systemic circulation	Peritoneal integrity Cavity organization and fluid dynamics Systemic vascular and immune constraints	Cavity-level and systemic exposure following upstream containment failure	Peritoneal carcinomatosis and distant metastatic disease

## Data Availability

The original contributions presented in this study are included in the article. Further inquiries can be directed to the corresponding authors.
